# Evaluation of Lyso-Gb1 as a biomarker for Gaucher disease treatment outcomes using data from the Gaucher Outcome Survey

**DOI:** 10.1186/s13023-024-03444-y

**Published:** 2025-01-29

**Authors:** Ari Zimran, Shoshana Revel-Vilk, Tama Dinur, Majdolen Istaiti, Jaco Botha, Elena Lukina, Pilar Giraldo, Patrick Deegan, Stephan vom Dahl

**Affiliations:** 1 Gaucher Unit, The Eisenberg R & D Authority, Shaare Zedek Medical Center, Jerusalem, Israel; 2 Faculty of Medicine, Hebrew University, Jerusalem, Israel; 3 Takeda Pharmaceuticals International AG, Zurich, Switzerland; 4 Department of Orphan Diseases, National Research Center for Hematology, Moscow, Russia; 5 Translational Research Unit, IIS Aragon, Zaragoza, Spain; 6 CIBER de Enfermedades Raras, IIS Aragon, Zaragoza, Spain; 7 Department of Medicine, Addenbrookes Hospital, University of Cambridge, Cambridge, UK; 8 Department of Gastroenterology, Hepatology and Infectious Diseases, University Hospital, Heinrich- Heine University, Düsseldorf, Germany

**Keywords:** Lyso-Gb1, Glucosylsphingosine, Gaucher disease, Biomarker

## Abstract

**Background:**

Patients with Gaucher disease (GD) require continual monitoring; however, lack of specific disease biomarkers was a significant challenge in the past. Glucosylsphingosine (lyso-Gb1) has been shown to be a reliable, key, specific, and sensitive biomarker for diagnosis, prognosis, and treatment response in clinical studies of patients with GD. We evaluated the change in lyso-Gb1 concentration over time following enzyme replacement therapy in patients with confirmed GD using real-world data from the Gaucher Outcome Survey disease registry.

**Methods:**

Data for patients aged ≥ 18 years with a confirmed diagnosis of GD and at least two lyso-Gb1 assessments were analyzed retrospectively. Patients were stratified by treatment status at baseline (time of first lyso-Gb1 assessment). Lyso-Gb1 concentrations were measured from dried blood spot (DBS) samples by Centogene AG. Assessments included change in lyso-Gb1 concentration, hemoglobin concentration, platelet counts, and spleen and liver volume from baseline to the last lyso-Gb1 assessment.

**Results:**

Of 2007 patients enrolled in the Gaucher Outcome Survey as of February 25, 2022, 435 met the inclusion criteria and were included in the study: 318 treated (‘all treated’; 277 receiving treatment at baseline, 41 treatment naive at baseline), 38 receiving treatment at baseline who stopped treatment before the last lyso-Gb1 assessment, and 79 untreated. Lyso-Gb1 concentrations decreased from baseline to the last lyso-Gb1 assessment for all treated patients (median change − 8.6 ng/mL), and increased for untreated patients (median change 25.0 ng/mL) and those who stopped treatment (median change 19.5 ng/mL). Decreases were greater for all treatment-naive than previously treated patients (median change − 120.5 vs. − 3.3 ng/mL) and for velaglucerase alfa–treated patients vs. the overall treated cohort (–32.6 vs. − 8.6 ng/mL). Small improvements in hemoglobin concentrations, platelet counts, and spleen volume were observed for treated patients but not untreated/stopped treatment cohorts.

**Conclusions:**

In this study, changes in lyso-Gb1 concentrations from DBS were reflective of responses to enzyme replacement therapy initiation or withdrawal in most patients. These findings confirm that the use of DBS samples for routine monitoring of lyso-Gb1 concentrations in patients with GD is feasible in real-world settings and may be useful to assess treatment response.

**Supplementary Information:**

The online version contains supplementary material available at 10.1186/s13023-024-03444-y.

## Background

Gaucher disease (GD) is a rare autosomal recessive lysosomal storage disorder caused by mutations in the glucocerebrosidase gene (*GBA1*). Subsequent deficient activity of the enzyme β-glucocerebrosidase (GCase) (OMIM# 230800/230900/231000) results in the accumulation of glucocerebroside (also called glucosylceramide; Gb1) in the lysosomes of cells of the monocyte-macrophage system [1–4]. Affected cells are transformed into Gaucher cells, which accumulate in the spleen, liver, and bone marrow, giving rise to multisystemic clinical manifestations including anemia, thrombocytopenia, hepatomegaly, splenomegaly, and bone abnormalities, hallmarks of type 1 GD, the most common form of GD [4]. Pulmonary and renal involvement are less common, whereas characteristic neurological involvement defines types 2 and 3 GD and is absent in type 1 GD [4]. A recent systematic review and meta-analysis estimates the birth prevalence of GD as 1.5 cases per 100,000 live births [5], although a higher frequency has been estimated (of approximately 1 in 850) among the Ashkenazi Jewish population [1].

The most common approach for GD diagnosis is the determination of GCase activity in peripheral blood cells in conjunction with DNA mutation analysis of the *GBA1* gene [6]. However, the requirement to ship fresh blood samples to one of the few specialist laboratories equipped for the assessment of GCase activity has been a barrier to GD diagnosis [6]. An alternative sample type, dried blood spot (DBS), has several practical advantages over conventional blood sampling, including storage at room temperature and shipping by regular mail [7]. DBS analysis can accurately detect glucosylsphingosine (lyso-Gb1), the deacylated form of glucocerebroside, a key pathogenic biomarker for GD [1, 6, 8, 9], with similar discriminatory utility as plasma sample analysis [10].

Elevated concentrations of lyso-Gb1 have been detected in blood and plasma samples of patients with GD compared with healthy controls and in patients with other lysosomal storage disorders [11, 12]. Lyso-Gb1 concentrations have also been shown to correlate with disease severity [6, 13], and to be reflective of response to GD treatment [8, 10, 14–16]. Higher concentrations of lyso-Gb1 in blood plasma have been found in patients with the c.1448T > C (L444P, now referred to as L483P) genetic variant, which is associated with severe disease, than in those with the c.1226A > G (N370S, now referred to as N409S) variant, which is associated with a milder disease course [12, 17]. Furthermore, 16 of 17 studies evaluated in a systematic review reported substantial decreases in lyso-Gb1 concentrations following enzyme replacement therapy (ERT) and substrate reduction therapy compared with untreated patients with GD [18], and decreases in lyso-Gb1 concentrations have been associated with improvements in hematologic and visceral parameters [16, 19–21]. Efforts to date to evaluate lyso-Gb1 as a predictive biomarker have, however, been constrained by small patient sample sizes and short durations of patient follow-up [11, 12, 16, 22].

The availability of more than 10 years of data from patients with GD enrolled in the Gaucher Outcome Survey (GOS), a registry for patients with confirmed diagnoses of GD, provides an opportunity for long-term analysis of patients who have received GD-specific treatments in a real-world setting [23–26]. The aim of this study was to evaluate changes in lyso-Gb1 concentrations over time in ERT-treated patients with confirmed GD in relation to treatment and clinical outcomes using clinical data captured by the GOS registry.

## Methods

### Study design

The GOS, established in 2010 by Shire, a Takeda company, is an international disease-specific registry for patients with a confirmed biochemical or genetic diagnosis of GD, regardless of treatment status or type of treatment received (ClinicalTrials.gov, NCT03291223). Data are collected via web-based electronic case report forms during routine clinical practice and include a comprehensive range of real-world patient characteristics and clinical outcomes [27].

Written informed consent is required for participation in the GOS. Consent, or assent where appropriate, is obtained from a parent or legal guardian for patients aged < 18 years (< 16 years in the United Kingdom). The study was conducted in accordance with relevant global and local regulations and best practice and with Good Pharmacoepidemiological Practice, Good Research for Comparative Effectiveness principles, and the principles of the International Conference on Harmonization Good Clinical Practice guidelines.

This retrospective analysis of the GOS data was conducted in Israel, Austria, Poland, and the United Kingdom between July 2, 2014, up to February 25, 2022, and included data from centers participating in the GOS with available lyso-Gb1 data evaluated by Centogene AG on DBS samples.

### Patients

**Fig. 1:**
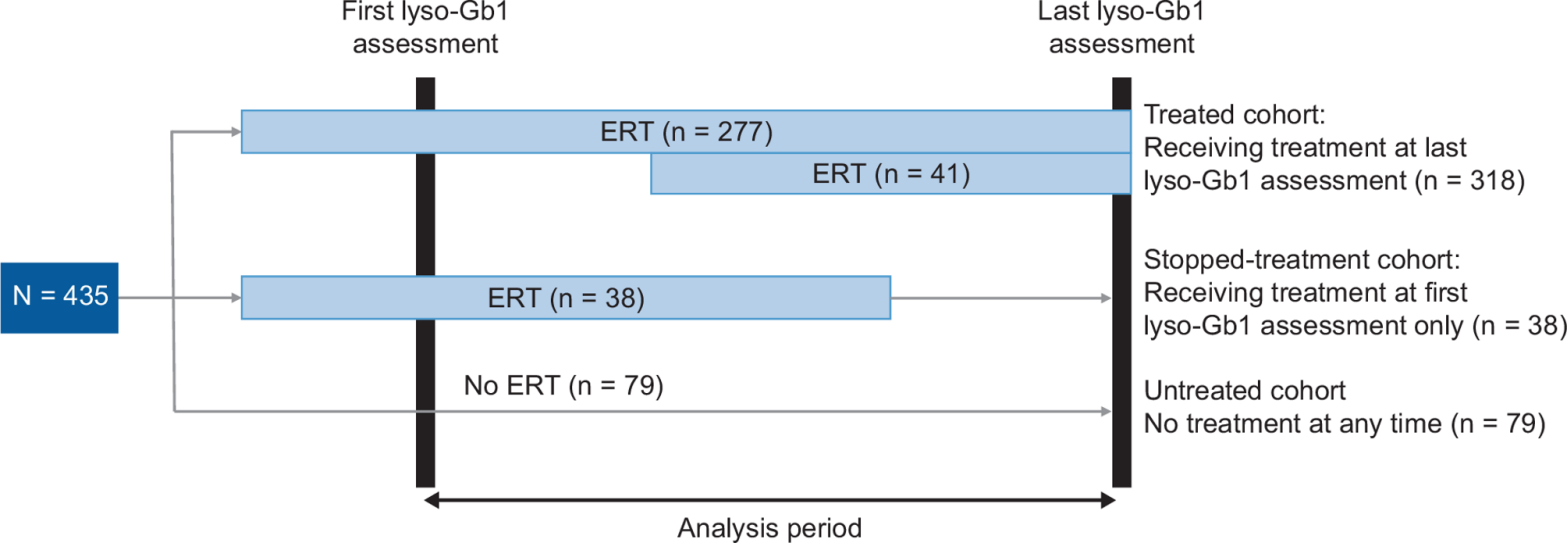
Patient stratification. Baseline was defined as the time of a patient’s first glucosylsphingosine (lyso-Gb1) assessment. Study duration was defined as the time between the first and last lyso-Gb1 assessments for each individual. ERT, enzyme replacement therapy

Data for patients aged ≥ 18 years on July 2, 2014, who had at least two lyso-Gb1 assessments were included in this analysis. Patients were stratified by treatment status (all treated, untreated, or stopped treatment) relative to the time of their first lyso-Gb1 assessment, defined as the start of the analysis period (baseline) (Fig. 1). The end of the analysis period was defined as the last lyso-Gb1 assessment. The untreated cohort included patients who received no ERT treatment prior to or during the analysis period; the stopped treatment cohort included patients who discontinued ERT treatment before the last lyso-Gb1 assessment; and the all treated cohort included all patients who were receiving ERT treatment at the time of the last lyso-Gb1 assessment. The all treated cohort was further divided into two subgroups based on treatment status at baseline (time of the first lyso-Gb1 assessment): (1) those who received treatment at the first lyso-Gb1 assessment and remained on ERT treatment to the last lyso-Gb1 assessment; and (2) patients who started ERT treatment after the first lyso-Gb1 assessment and remained on treatment at the last lyso-Gb1 assessment. Treatment-naive patients were defined as those patients who had not received treatment ≥ 12 months prior to the start of the study. Further sub-analyses were carried out for patients who were treated with velaglucerase alfa only.

### Assessments

Lyso-Gb1 concentrations analyzed by Centogene AG after July 2, 2014, using liquid chromatography–mass spectrometry analysis of DBS samples as described by Cozma et al. [8], were included in this evaluation. Assessments included change in median lyso-Gb1 concentration by treatment group during the analysis period (from the first to last lyso-Gb1 assessment); the proportion of patients with increases or decreases of ≥ 10% (arbitrary threshold) or no change in lyso-Gb1 concentration during the analysis period; and change in hemoglobin concentrations, platelet count, spleen volume, and liver volume during the analysis period.

### Statistical analyses

Baseline was defined as the time of the first lyso-Gb1 assessment. Data for continuous variables were presented using descriptive statistics. For categorical variables, the number and percentage of patients in each category (including a missing category, if applicable) were reported. Percentages were calculated using the number of patients with available data as the denominator. Changes in lyso-Gb1 concentration and clinical parameters over time were additionally assessed using linear regression, where β represents the slope (rate of change). To account for the non-linear response of lyso-Gb1 to treatment, only patients with more than three lyso-Gb1 assessments were included in linear regression models to estimate changes in lyso-Gb1 concentrations. Similarly, models estimating changes in clinical parameters only included patients with three or more assessments of that parameter. For the assessment of hematologic parameters, hemoglobin values < 50 and > 400 g/L and platelet counts > 600 × 10^9^/L were classed as biologically improbable outliers and were excluded from the analyses. Statistical analyses were performed using SAS version 9.4 (SAS Institute, Cary, NC, USA).

## Results

### Patient characteristics

Of 2007 adults enrolled in the GOS as of February 25, 2022, 435 fulfilled the inclusion criteria (aged ≥ 18 years with at least two lyso-Gb1 assessments) and were included in this study. Of these, 318 (73.1%) were receiving ERT at the last lyso-Gb1 assessment (all treated cohort: 277 [87.1%] started treatment before the first lyso-Gb1 assessment; 41 [12.9%] were treatment naive at the time of the first lyso-Gb1 assessment), 38 (8.7%) were receiving treatment at the time of the first lyso-Gb1 assessment but stopped treatment before the last lyso-Gb1 assessment, and 79 (18.2%) remained untreated throughout the analysis period.

Age at baseline and sex distribution were similar across the all treated, untreated, and stopped treatment cohorts (Table 1). The c.1226A > G (N370S/N409S) homozygous *GBA1* mutation was recorded for 74.7% and 73.7% of untreated and stopped treatment patients, respectively, compared with 49.4% of treated patients. The duration of treatment prior to the first lyso-Gb1 assessment was greater for 277 patients who remained on treatment (all treated cohort; median [range] 14.3 [0.1–29.4] years) compared with those who stopped treatment during the analysis period (median [range] 7.7 [0.3–22.0] years). The median time between the first and last lyso-Gb1 assessment was just over 5 years for the treated and stopped treatment cohorts and 3.9 years for untreated patients.

**Table 1:** Patient demographics and characteristics at baseline (time of the first lyso-Gb1 assessment)

	Untreated (*n* = 79)	All treated (*n* = 318)	Stopped treatment (*n* = 38)
Age at first lyso-Gb1 assessment, y			
Mean (SD)	44.3 (15.36)	44.2 (15.84)	41.8 (13.75)
Median (range)	44.3 (19.6–79.2)	41.6 (17.6–88.0)	40.0 (19.5–75.8)
Age on July 2, 2014, y			
Mean (SD)	43.6 (15.48)	44.0 (16.02)	42.2 (13.67)
Median (range)	43.5 (18.5–80.2)	42.3 (18.3–87.9)	39.4 (21.0–77.0)
Sex, n (%)			
Male	33 (41.8)	138 (43.4)	17 (44.7)
Variants in *GBA1* or disease type, n (%)			
c.1226A > G (N370S/N409S)/c.1226 A> G (N370S/N409S)	59 (74.7)	157 (49.4)	28 (73.7)
c.1226A > G (N370S/N409S)/other	17 (21.5)	115 (36.2)	8 (21.1)
c.1448T > C (L444P/L483P)	3 (3.8)	23 (7.2)	2 (5.3)
Not available/unknown	0 (0)	23 (7.2)	0 (0)
Total splenectomy, n (%)	9 (11.4)	59 (18.6)	4 (10.5)
Duration of treatment prior to first lyso-Gb1 assessment, y			
n	0	277	30
Mean (SD)	–	13.3 (7.5)	9.1 (6.9)
Median (range)	–	14.3 (0.1–29.4)	7.7 (0.3–22.0)
Time between first and last lyso-Gb1 assessment, y			
Mean (SD)	4.1 (1.7)	4.7 (1.9)	4.9 (1.4)
Median (range)	3.9 (1.1–7.5)	5.1 (0.5–7.5)	5.1 (2.4–6.9)
Hemoglobin concentration, g/L			
n	76	312	38
Mean (SD)	132.6 (14.84)	133.9 (15.17)	128.2 (19.12)
Median (range)	132.5 (87.0–168.0)	134.0 (96.0–173.0)	131.0 (89.0–161.0)
Platelet count, × 10^9^/L			
n	75	312	38
Mean (SD)	156.2 (81.95)	160.1 (83.30)	124.6 (60.08)
Median (range)	139.0 (30.0–430.0)	146.0 (17.0–476.0)	130.5 (10.0–331.0)
Liver size, MoN			
n	64	237	35
Mean (SD)	1.1 (0.20)	1.1 (0.22)	1.2 (0.22)
Median (range)	1.1 (0.5–1.7)	1.1 (0.5–2.0)	1.1 (0.8–1.7)
Spleen size, MoN			
n	56	200	32
Mean (SD)	6.4 (1.84)	8.1 (3.20)	8.3 (3.30)
Median (range)	6.1 (3.8–13.2)	7.4 (3.2–23.1)	8.0 (3.5–17.4)

Genetic information was available for 412 (94.7%) patients overall. For hematologic outcomes, hemoglobin concentrations were available for 426 (97.9%) patients and platelet counts were available for 425 (97.7%). For visceral outcomes, liver and spleen volumes were available for 336 (77.2%) and 288/363 non-splenectomized (79.3%) patients, respectively

Lyso-Gb1, glucosylsphingosine; MoN, multiple of normal; SD, standard deviation

Liver MoN of 1.0 was defined as 2.5% of body weight and spleen MoN of 1.0 was defined as 0.2% of body weight

### Change in lyso-Gb1 over time

Median lyso-Gb1 concentrations at baseline (first lyso-Gb1 assessment) were 92.6 (range 3.0–1140.0) ng/mL in the all treated cohort, 109.0 (7.9–660.0) ng/mL in untreated patients, and 100.3 (15.5–506.0) ng/mL in patients who stopped ERT treatment (Fig. S1). In the all treated cohort, baseline lyso-Gb1 concentrations were higher for patients who were treatment naive (224.0 [67.1–1140.0] ng/mL, *n* = 41) than for those already receiving treatment at baseline (78.2 [3.0–898.0] ng/mL, *n* = 277).

**Fig. 2:**
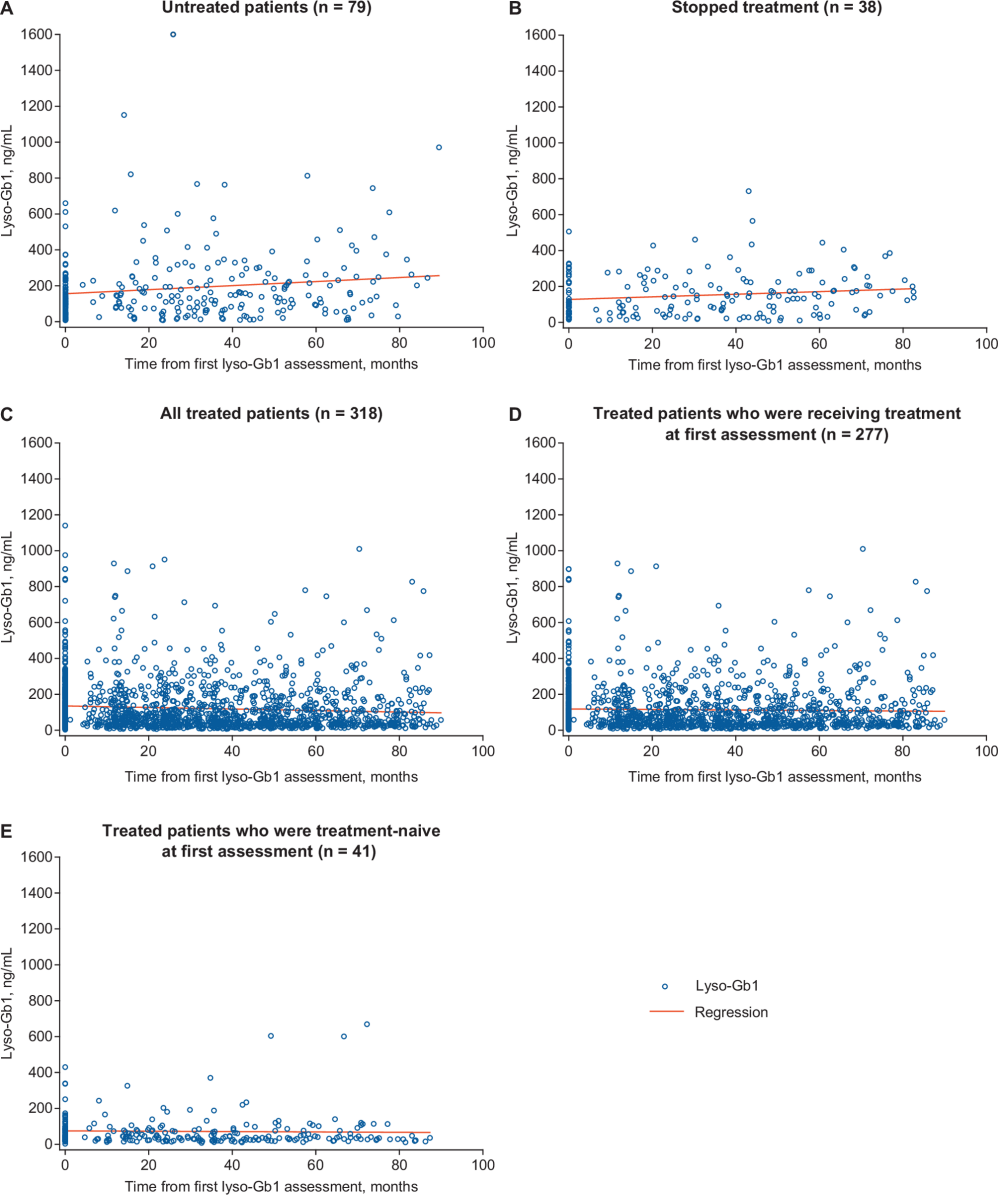
Scatter plots showing distribution of individual glucosylsphingosine (lyso-Gb1) levels from first to last assessment by treatment group: (**A**) untreated patients (*n* = 79); (**B**) patients who stopped treatment (*n* = 38); (**C**) all treated patients (*n* = 318); (**D**) treated patients who were receiving treatment at first assessment (*n* = 277); and (**E**) treated patients who were treatment-naive at first assessment (*n* = 41). Lyso-Gb1 levels < 6.8 ng/ml are considered normal [6]

Decreases in lyso-Gb1 were observed over the follow-up period for most patients in the all treated cohort. Patients who were treatment naive at the first lyso-Gb1 assessment had a median (range) decrease of 120.5 (–427.0 to 349) ng/mL, *n* = 41) and lyso-Gb1 concentrations decreased by ≥ 10% (arbitrary threshold) in 36 of 41 (87.8%) patients (median decrease of 138.8 ng/mL) (Fig. S1). Patients receiving treatment at both the first and last lyso-Gb1 assessments (*n* = 277) had a median (range) decrease of 3.3 (–729.6 to 697.0) ng/mL, with decreases of ≥ 10% occurring in 132 of 277 (47.7%) patients (median decrease of − 46.0 ng/mL). In contrast, increases were observed for untreated patients and those who stopped treatment prior to the last lyso-Gb1 assessment (Fig. S1). Lyso-Gb1 concentrations increased by ≥ 10% in 56 of 79 (70.9%) untreated patients (median increase 54.8 ng/mL) and in 22 of 38 (57.9%) patients who stopped treatment (median increase 75.5 ng/mL). Scatter plots depicting the distribution of individual lyso-Gb1 concentrations from first to last assessment in the various treatment groups are presented in Fig. 2.

### Patients treated with velaglucerase alfa

Among the 318 patients who received treatment, 78 (24.5%) received velaglucerase alfa as their only form of ERT. Of these, 50 patients were receiving treatment at baseline and 28 were treatment naive at baseline. The median (range) velaglucerase alfa dose was similar for those receiving treatment at baseline (30.0 [15.0–84.6] U/kg) to those who started treatment after the baseline assessment (30.0 [15.0–60.0] U/kg). Dose reductions occurred between the first and last lyso-Gb1 assessments for some patients (Table 2).

**Table 2:** Velaglucerase alfa dose in patients treated with velaglucerase alfa only

	Receiving treatment at first lyso-Gb1 assessment (*n* = 49)		Treatment naive at first lyso-Gb1 assessment (*n* = 28)	
	First dose	Last dose	First dose	Last dose
Dose, U/kg				
Mean (SD)	32.4 (20.54)	27.9 (17.77)	39.6 (20.09)	35.4 (19.2)
Median (range)	30.0 (15.0–84.6)	30.0 (15.0–84.6)	30.0 (15.0–60.0)	30.0 (15.0–60.0)
Dose category, n (%)				
≤ 15 U/kg	24 (49.0)	24 (49.0)	8 (28.6)	10 (35.7)
> 15 to ≤ 30 U/kg	8 (16.3)	17 (34.7)	7 (25.0)	7 (25.0)
> 30 to ≤ 45 U/kg	3 (6.1)	1 (2.0)	0 (0)	2 (7.1)
> 45 to ≤ 60 U/kg	13 (26.5)	5 (10.2)	13 (46.4)	9 (32.1)
> 60 U/kg	1 (2.0)	2 (4.1)	0 (0)	0 (0)

Data show subgroups of patients from the all treated group treated with velaglucerase alfa only. Dose was bi-weekly or every other week, referring to calculated bi-weekly dosing interval

Lyso-Gb1, glucosylsphingosine; SD, standard deviation

**Fig. 3:**
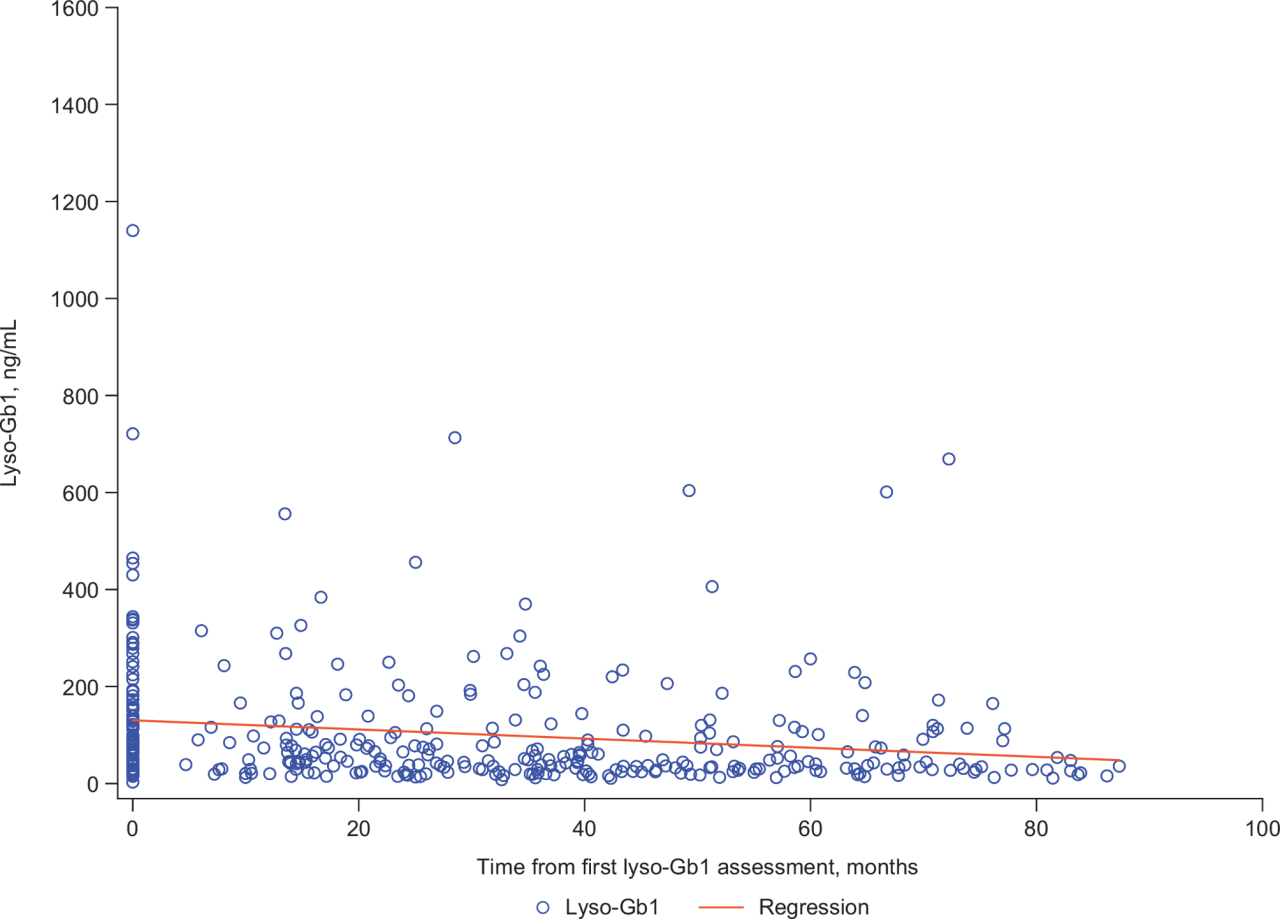
Scatter plot showing distribution of individual glucosylsphingosine (lyso-Gb1) levels from first to last assessment in patients treated with velaglucerase alfa only (*n* = 78). Lyso-Gb1 levels < 6.8 ng/ml are considered normal [6]

Overall, decreases in lyso-Gb1 concentrations over the assessment period were numerically greater for 78 patients treated with velaglucerase alfa (–32.6 ng/mL) than for 318 patients treated with any ERT (treated cohort, inclusive of those receiving velaglucerase alfa; − 8.6 ng/mL), despite similar baseline values (Fig. S2). Among the 240 patients treated with any ERT excluding velaglucerase alfa, the decrease in lyso-Gb1 concentrations was − 2.5 ng/mL. A scatter plot showing the distribution of individual lyso-Gb1 concentrations from first to last assessment in patients treated with velaglucerase alfa only is presented in Fig. 3.

Among the 41 patients in the all treated cohort (any ERT) who were treatment naive at baseline, a decrease in lyso-Gb1 concentration of − 120.5 ng/mL was observed (Fig. S1). In patients treated with velaglucerase alfa only, those who were treatment naive at baseline (*n* = 28) had a decrease in lyso-Gb1 concentration of − 138.8 ng/mL, while a decrease of − 12.8 ng/mL was observed among the 50 patients who were treated with velaglucerase alfa at baseline (Fig. S2). All patients who were velaglucerase alfa naive at baseline had decreased (96.4%) or stable (3.6%) lyso-Gb1 concentrations over the assessment period. Of those receiving velaglucerase alfa at the first assessment, 33 (66.0%) patients showed decreased or stable lyso-Gb1, whereas lyso-Gb1 concentrations increased by ≥ 10% from baseline for 17 (34.0%) patients over the assessment period (Fig. S2). However, baseline values and absolute changes in lyso-Gb1 were small (median [Q1–Q3] 39.5 [20–57.7] ng/mL at baseline and 17.8 [13.5–45.3] ng/mL change from baseline to last assessment), with the exception of one patient who had an increase in lyso-Gb1 from 339 to 669 ng/mL over 9.2 years of follow-up following a break in treatment between February 2016 and January 2022. Hematologic outcomes remained consistent over the assessment period for all 17 patients. Median (Q1–Q3) hemoglobin concentrations were 132 (116–139) g/L at baseline and 128 (121–132) g/L at the last assessment, and platelet counts were 213 (144–232) × 10^9^/L at baseline and 177 (144–220) × 10^9^/L at the last assessment.

### Lyso-Gb1 level by disease type

Analysis of individual lyso-Gb1 levels (at last assessment) by disease genotype indicated a higher range of values in patients with type 1 GD heterozygous for N370S compared to patients with type 1 GD homozygous for N370S or patients with type 3 (neuronopathic) GD (Fig. S3).

### Change in lyso-Gb1 vs. clinical parameters

The cohort of 41 treatment-naive patients had a significant annual decrease in lyso-Gb1 concentration (β = − 25.9 [95% CI − 34.6 to − 17.1]) after treatment initiation, as well as small but significant improvements in hemoglobin concentrations (β = 1.5 [0.8–2.1]; 51 samples were excluded as outliers, leaving 5095 samples for inclusion), platelet counts (β = 10.0 [7.8–12.2]; 33 samples were excluded as outliers, leaving 5082 samples for inclusion), and spleen volume (non-splenectomized patients only; β = − 0.4 [–0.5 to − 0.2]), and no change in liver volume (β = − 0.01 [–0.02 to 0.0]) (Table 3). In contrast, the cohort of 79 untreated patients had a significant annual increase in lyso-Gb1 concentration from the first to last lyso-Gb1 assessment (β = 11.9 [5.2–18.7]), with no marked changes in hematologic or visceral outcomes (Table 3). However, no significant correlations between lyso-Gb1 concentrations and clinical parameters were observed (R^2^ values range 0.0011–0.4550), likely owing to high inter- and intra-patient variation and the small sample size. Graphical explorations of the relationships between lyso-Gb1 level and selected clinical outcomes (hemoglobin level, platelet count, and spleen volume) are presented in Fig. S4.

**Table 3:** Estimated annual rate of change in lyso-Gb1 and clinical parameters in treatment naive ERT initiated and untreated patients

	Treatment naive (*n* = 41)	Untreated (*n* = 79)
Lyso-Gb1 concentrations, ng/mL, β (95% CI)	–25.9 (–34.6 to − 17.1)	11.9 (5.2–18.7)
Hemoglobin, g/L, β (95% CI)	1.5 (0.8–2.1)	0.1 (–0.4 to 0.6)
Platelet count, x 10^9^/L, β (95% CI)	10.0 (7.8–12.2)	0.8 (–0.8 to 2.3)
Spleen size, MoN, β (95% CI)	–0.4 (–0.5 to − 0.2) [*n* = 36]	–0.04 (–0.2 to 0.1) [*n* = 66]
Liver size, MoN, β (95% CI)	–0.01 (–0.02 to 0.0) [*n* = 39]	0.01 (0.0–0.01) [*n* = 75]

Liver MoN of 1.0 was defined as 2.5% of body weight and spleen MoN of 1.0 was defined as 0.2% of body weight. Models to estimate changes in the above parameters only included patients with three or more assessments of that parameter. Estimates for change in spleen size included non-splenectomized patients only

β, estimated slope; CI, confidence interval; lyso-Gb1, glucosylsphingosine; MoN, multiple of normal

## Discussion

In this evaluation of data from the GOS, changes in lyso-Gb1 concentrations measured from DBS samples were found to be reflective of ERT treatment status in most patients. Treatment-naive patients (i.e., those with a “true” baseline assessment of lyso-Gb1 prior to ERT initiation) had both higher lyso-Gb1 concentrations at the baseline assessment and numerically greater decreases in lyso-Gb1 concentration with treatment than patients who were already receiving GD-specific treatment at the baseline assessment, consistent with previously published findings [6, 8, 9, 16, 28]. These results suggest that patients already receiving treatment at baseline were likely to have experienced decreases in lyso-Gb1 concentrations prior to the first assessment in this analysis. Additionally, numerically larger decreases in lyso-Gb1 concentration were observed for those treated with velaglucerase alfa compared with the overall treated cohort including those treated with any ERT excluding velaglucerase alfa, consistent with a separate study that evaluated lyso-Gb1 concentrations on DBS samples [29]. These results are supportive of a proposed small “booster effect” of this ERT, thought to be a result of its wild-type human sequence and superior internalization into human macrophages compared with other ERTs [30, 31]. Increases in lyso-Gb1 concentrations observed among untreated patients and those who stopped treatment are similarly in line with previous findings that lyso-Gb1 concentrations increase after the cessation of treatment [12, 16], and may indicate the utility of lyso-Gb1 to detect disease exacerbations during drug holidays, particularly in patients who have a chitotriosidase null mutation.

Previous work has explored the value of lyso-Gb1 as a prognostic and disease-monitoring biomarker [11, 12, 16, 22]. In our study, significant decreases in lyso-Gb1 concentrations and spleen volume, and significant increases in hemoglobin concentrations and platelet counts were observed in treatment-naive patients after initiation of GD-specific treatment, in line with previous findings [8, 16, 28]. For untreated patients, a significant increase in lyso-Gb1 concentration was observed between baseline and last available assessments, with no corresponding changes in clinical parameters. The greater magnitude of change in lyso-Gb1 compared with clinical outcomes suggests effects on this biomarker might precede clinically significant events.

Considerable inter-patient variation was observed. More than a third of treated patients had an increased lyso-Gb1 concentration of ≥ 10% from baseline to last assessment—most of whom (115/118 [97.5%]) had initiated ERT prior to the first lyso-Gb1 assessment—compared with approximately two-thirds of untreated patients or those who stopped treatment. However, absolute changes were small for most patients, and were not reflected by changes in hemoglobin concentrations or platelet counts. This variation may be explained by patient and disease-related factors; previous studies have identified factors such as age, circadian rhythm, effects of nutrition and/or physical activity, or effects of coexisting pathological conditions as potential explanations for a high variability in lyso-Gb1 measurements [8, 28]. This variability and overlap between treated/untreated patients underline the importance of longitudinal measurements to accurately assess treatment outcomes and disease progression. A responder analysis, with stratification by factors such as age at symptom onset, age at diagnosis, timing of ERT, disease severity, clinical parameters at baseline, and treatment variation could be beneficial to further understand the variability of lyso-Gb1 responses in individual patients.

Our analysis provides important information on trends of lyso-Gb1 change with respect to treatment. The use of the GOS, a rare-disease registry, affords the opportunity to collect longitudinal data from a larger and more varied patient cohort than clinical trials, and provides insights into real-world treatment utilization and outcomes. However, certain limitations are inherent to the use of such registries. Although enrollment in the GOS is open to all patients with a confirmed diagnosis of GD, irrespective of treatment status or type, there is a potential for bias toward inclusion of velaglucerase alfa–treated patients in a registry sponsored by the manufacturer; velaglucerase alfa–treated patients may be overrepresented in this cohort, specifically in treatment-naive patients, which might in part explain the numerically greater decrease in lyso-Gb1 concentrations observed among these patients. Data are collected during routine clinical practice where the frequency of visits and type of assessments can vary considerably between patients, and the quality and quantity of the data depends on the input provided by multiple physicians and other users. As such, data may be incomplete or inconsistent. To mitigate this, biologically improbable outliers (i.e., hemoglobin values < 50 and > 400 g/L and platelet count values > 600 × 10⁹/L) were omitted from these analyses, although this may have resulted in the inadvertent exclusion of data from splenectomized patients. The measurement of lyso-Gb1 as a GD-specific biomarker is not standardized and can vary considerably between laboratories. To limit inter-laboratory variability, assessments of lyso-Gb1 using only the Centogene DBS Assay (Rostock, Germany) were included in this study; however, the exclusion of assessments using other assays limited the number of participants available for the analysis. Three-quarters of patients had the homozygous c.1226A > G (N370S/N409S) genotype, reflective of the high proportion of included patients from Israel. In addition, the study excluded children (aged < 18 years) owing to a lack of available data, further reducing the patient numbers for evaluation. Nevertheless, the disease characteristics (GD type, splenectomy status, liver and spleen volumes, hemoglobin level and platelet count) of our study population were generally similar to those reported for the overall GOS registry population [27]. 

## Conclusions

In this evaluation of data from the GOS, numerically larger decreases in lyso-Gb1 were observed in patients treated with velaglucerase alfa compared with other ERTs. Long-term monitoring of lyso-Gb1 concentrations using DBS from patients with GD in real-world clinical settings suggests changes in this biomarker were reflective of ERT treatment response in most patients, providing support for the utility of lyso-Gb1 measurements on DBS samples for routine monitoring of patients with GD. With consideration of the inherent limitations of real-world data, this study indicates that routine monitoring of lyso-Gb1 concentrations in clinical practice is feasible, although further research is required to understand the relationship between lyso-Gb1 and clinical parameters.

## Electronic supplementary material

Below is the link to the electronic supplementary material.

Supplementary Material 1: Fig. S1. Change in glucosylsphingosine (lyso-Gb1) level from the first to last assessment by treatment group.

Supplementary Material 2: Fig. S2. Change in glucosylsphingosine (lyso-Gb1) level by treatment group in patients treated with velaglucerase alfa only.

Supplementary Material 3: Fig. S3. Individual lyso-Gb1 levels at last assessment in treated and untreated patients categorized by Gaucher disease genotype.

Supplementary Material 4: Fig. S4. Exploratory plots of the relationship between lyso-Gb1 level at last assessment and: (A) hemoglobin level, (B) platelet count, (C) spleen volume, and (D) liver volume.

## Data Availability

The datasets, including the redacted study protocol, redacted statistical analysis plan, and individual participants’ data supporting the results reported in this article, will be made available within 3 months from initial request to researchers who provide a methodologically sound proposal. The data will be provided after its de-identification, in compliance with applicable privacy laws, data protection, and requirements for consent and anonymization.

## Abbreviations

CI: Confidence interval
DBS: Dried blood spot
ERT: Enzyme replacement therapy
Gb1: Glucocerebroside
GCase: Β-glucocerebrosidase
GD: Gaucher disease
GOS: Gaucher Outcome Survey
Lyso-Gb1: Glucosylsphingosine
MoN: Multiple of normal
SD: Standard deviation

## References

[1] Aerts J, Kuo CL, Lelieveld LT, et al. Glycosphingolipids and lysosomal storage disorders as illustrated by Gaucher disease. Curr Opin Chem Biol. 2019;53:204–15. 31783225 10.1016/j.cbpa.2019.10.006

[2] Ferraz MJ, Kallemeijn WW, Mirzaian M, et al. Gaucher disease and Fabry disease: new markers and insights in pathophysiology for two distinct glycosphingolipidoses. Biochim Biophys Acta. 2014;1841(5):811–25. 10.1016/j.bbalip.2013.11.004 24239767

[3] Hruska KS, LaMarca ME, Scott CR, Sidransky E. Gaucher disease: mutation and polymorphism spectrum in the glucocerebrosidase gene (GBA). Hum Mutat. 2008;29(5):567–83. 18338393 10.1002/humu.20676

[4] Zimran A, Elstein D. Gaucher disease and related lysosomal storage diseases. In: Kaushanksy K, Lichtman M, Prchal J, Levi MM, Press O, Burns L, Caligiuri M, editors. Williams hematology. 9th ed. New York, NY, USA: McGraw-Hill; 2016.

[5] Wang M, Li F, Zhang J, Lu C, Kong W. Global epidemiology of Gaucher disease: an updated systematic review and meta-analysis. J Pediatr Hematol Oncol. 2023;45(4):181–8. 35867706 10.1097/MPH.0000000000002506 PMC10115488

[6] Dinur T, Bauer P, Beetz C, et al. Gaucher disease diagnosis using lyso-Gb1 on dry blood spot samples: time to change the paradigm? Int J Mol Sci. 2022;23(3):1627. 35163551 10.3390/ijms23031627 PMC8835963

[7] Moat SJ, George RS, Carling RS. Use of dried blood spot specimens to monitor patients with inherited metabolic disorders. Int J Neonatal Screen. 2020;6(2):26. 33073023 10.3390/ijns6020026 PMC7422991

[8] Cozma C, Cullufi P, Kramp G, et al. Treatment efficiency in Gaucher patients can reliably be monitored by quantification of lyso-Gb1 concentrations in dried blood spots. Int J Mol Sci. 2020;21(13):4577. 32605119 10.3390/ijms21134577 PMC7369829

[9] Dinur T, Bauer P, Beetz C, et al. Contribution of glucosylsphingosine (lyso-Gb1) to treatment decisions in patients with gaucher disease. Int J Mol Sci. 2023;24(4):3945. 10.3390/ijms24043945 PMC9966520 36835356

[10] Saville JT, McDermott BK, Chin SJ, Fletcher JM, Fuller M. Expanding the clinical utility of glucosylsphingosine for Gaucher disease. J Inherit Metab Dis. 2020;43(3):558–63. 31707742 10.1002/jimd.12192

[11] Dekker N, van Dussen L, Hollak CE, et al. Elevated plasma glucosylsphingosine in Gaucher disease: relation to phenotype, storage cell markers, and therapeutic response. Blood. 2011;118(16):e118–27. 21868580 10.1182/blood-2011-05-352971 PMC3685900

[12] Rolfs A, Giese AK, Grittner U, et al. Glucosylsphingosine is a highly sensitive and specific biomarker for primary diagnostic and follow-up monitoring in Gaucher disease in a non-Jewish, Caucasian cohort of Gaucher disease patients. PLoS ONE. 2013;8(11):e79732. 10.1371/journal.pone.0079732 PMC3835853 24278166

[13] Curado F, Rosner S, Zielke S, et al. Insights into the value of Lyso-Gb1 as a predictive biomarker in treatment-naive patients with Gaucher Disease Type 1 in the LYSO-PROOF study. Diagnostics (Basel). 2023;13:17. 10.3390/diagnostics13172812 PMC10487050 37685353

[14] Arkadir D, Dinur T, Revel-Vilk S, et al. Glucosylsphingosine is a reliable response biomarker in Gaucher disease. Am J Hematol. 2018;93(6):E140–2. 29473199 10.1002/ajh.25074

[15] Malinova V, Poupetova H, Reboun M, et al. Long-term evaluation of biomarkers in the Czech Cohort of Gaucher patients. Int J Mol Sci. 2023;24:19. 10.3390/ijms241914440 PMC10572410 37833892

[16] Elstein D, Mellgard B, Dinh Q, et al. Reductions in glucosylsphingosine (lyso-Gb1) in treatment-naïve and previously treated patients receiving velaglucerase alfa for type 1 Gaucher disease: data from phase 3 clinical trials. Mol Genet Metab. 2017;122(1–2):113–20. 10.1016/j.ymgme.2017.08.005 28851512

[17] Mao XY, Burgunder JM, Zhang ZJ, et al. Association between GBA L444P mutation and sporadic Parkinson’s disease from Mainland China. Neurosci Lett. 2010;469(2):256–9. 20004703 10.1016/j.neulet.2009.12.007

[18] Revel-Vilk S, Fuller M, Zimran A. Value of glucosylsphingosine (lyso-Gb1) as a biomarker in Gaucher disease: a systematic literature review. Int J Mol Sci. 2020;21(19):7159. 32998334 10.3390/ijms21197159 PMC7584006

[19] Mirzaian M, Wisse P, Ferraz MJ, et al. Mass spectrometric quantification of glucosylsphingosine in plasma and urine of type 1 Gaucher patients using an isotope standard. Blood Cells Mol Dis. 2015;54(4):307–14. 10.1016/j.bcmd.2015.01.006 25842368

[20] Murugesan V, Chuang WL, Liu J, et al. Glucosylsphingosine is a key biomarker of Gaucher disease. Am J Hematol. 2016;91(11):1082–9. 27441734 10.1002/ajh.24491 PMC5234703

[21] Franco M, Reihani N, Marin M, et al. Effect of velaglucerase alfa enzyme replacement therapy on red blood cell properties in Gaucher disease. Am J Hematol. 2017;92(9):E561–3. 28621801 10.1002/ajh.24816

[22] Ferraz MJ, Marques AR, Appelman MD, et al. Lysosomal glycosphingolipid catabolism by acid ceramidase: formation of glycosphingoid bases during deficiency of glycosidases. FEBS Lett. 2016;590(6):716–25. 26898341 10.1002/1873-3468.12104

[23] Ben Turkia H, Gonzalez DE, Barton NW, et al. Velaglucerase alfa enzyme replacement therapy compared with imiglucerase in patients with Gaucher disease. Am J Hematol. 2013;88(3):179–84. 10.1002/ajh.23382 23400823

[24] Gonzalez DE, Turkia HB, Lukina EA, et al. Enzyme replacement therapy with velaglucerase alfa in Gaucher disease: results from a randomized, double-blind, multinational, phase 3 study. Am J Hematol. 2013;88(3):166–71. 23386328 10.1002/ajh.23381

[25] Zimran A, Altarescu G, Philips M, et al. Phase 1/2 and extension study of velaglucerase alfa replacement therapy in adults with type 1 Gaucher disease: 48-month experience. Blood. 2010;115(23):4651–6. 10.1182/blood-2010-02-268649 20299511

[26] Zimran A, Pastores GM, Tylki-Szymanska A, et al. Safety and efficacy of velaglucerase alfa in Gaucher disease type 1 patients previously treated with imiglucerase. Am J Hematol. 2013;88(3):172–8. 23339116 10.1002/ajh.23383 PMC3586535

[27] Zimran A, Belmatoug N, Bembi B, et al. Demographics and patient characteristics of 1209 patients with Gaucher disease: descriptive analysis from the Gaucher Outcome Survey (GOS). Am J Hematol. 2018;93(2):205–12. 10.1002/ajh.24957 PMC5814927 29090476

[28] Hurvitz N, Dinur T, Becker-Cohen M, et al. Glucosylsphingosine (lyso-Gb1) as a biomarker for monitoring treated and untreated children with Gaucher disease. Int J Mol Sci. 2019;20(12):3033. 10.3390/ijms20123033 PMC6627663 31234327

[29] Dinur T, Grittner U, Revel-Vilk S, et al. Impact of long-term enzyme replacement therapy on glucosylsphingosine (lyso-Gb1) values in patients with type 1 Gaucher disease: statistical models for comparing three enzymatic formulations. Int J Mol Sci. 2021;22(14):7699. 34299318 10.3390/ijms22147699 PMC8307068

[30] Elstein D, Altarescu G, Maayan H, et al. Booster-effect with velaglucerase alfa in patients with Gaucher disease switched from long-term imiglucerase therapy: early access program results from Jerusalem. Blood Cells Mol Dis. 2012;48(1):45–50. 10.1016/j.bcmd.2011.09.009 22047948

[31] Revel-Vilk S, Szer J, Mehta A, Zimran A. How we manage Gaucher disease in the era of choices. Br J Haematol. 2018;182(4):467–80. 29808905 10.1111/bjh.15402

